# Development of blood transfusion service in Sultanate of Oman

**DOI:** 10.4103/0973-6247.59390

**Published:** 2010-01

**Authors:** Sanmukh R. Joshi, Shahnaz N. Shah Al-Bulushi, Thamina Ashraf

**Affiliations:** *Institute of Health Sciences and Department of Blood Services, Ministry of Health, Muscat, Oman*

**Keywords:** Blood transfusion services, voluntary blood donation, national blood transfusion services, Sultanate of Oman

## Abstract

**Background:**

Sultanate of Oman is geographically situated in south-west of Asia, having common borders on western side by the land with United Arab Emirates, Saudi Arabia and Yemen and with the Arabian Sea and the Gulf of Oman in the east and the north respectively. The country enjoys one of the best health care facilities including blood transfusion services in the region.

**Study design:**

Information was collected through informal personal interviews, digging out the past records, and the report presentations at various forums.

**Results:**

A modest start by providing blood units through import, the country is now self-reliant on procuring blood units from voluntary non-remunerate blood donors within the sultanate. A steady growth of blood banks is witnessed in every aspect of blood banking including blood collection, blood processing and supply. Various modalities are adapted in promoting voluntary blood donation programme.

**Conclusion:**

Sultanate of Oman has created one of the best blood transfusion services in the region in providing safe blood for transfusion through voluntary donation, a use of blood components and irradiating blood products.

Sultanate of Oman is relatively a young country. The history of blood banking in Oman dates back with the history of the medical services established in mid 70s. Because of an unavailability of blood donors locally, blood was imported during those days. On an average, some 150 blood units were imported fortnightly, mainly from USA. Blood units were just stored and issued to the hospital whenever asked for. As these units were received as “finished” products, no tests were carried out again for transfusion transmitted infections on arrival in Oman. The blood units were issued for transfusion after necessary cross-match with the recipient. With an advent of newly emerged transfusion transmitted infections and its apprehension, particularly for the HIV, it was felt by the Ministry to test and confirm the imported units for its transfusion safety. So, in the year1984, the country had initiated the screening program on the imported blood for Hepatitis B (HBsAg) and HIV (Anti-HIV), no matter whether they already screened at the point of origin. This became the first attempt to impose safety measures on blood used for transfusion in the country. Although this confirmative testing facility was outsourced to the laboratories in the UK/Kuwait with the help of the WHO, indigenous testing was carried out from 1989 first at the Microbiology department of the University Hospital and then a year later at the Public Health Laboratory of the Ministry of Health in Oman.

## No More Import

The Ministry of Health decided to rely on the indigenous blood donor resources to provide safe blood to the national requirement. Efforts were put in this direction in early 1990. With modest efforts, we could organize two to three blood donation drives per month to get some useful blood units from the local communities. As an incentive, we used to pay a sum of RO 10/- (about US $25 at the current rate of exchange!). We were aware of the nuisance of such paid blood donations but we considered it as a motivating factor to bring the people in forefront of donation, particularly when the idea to donate blood was naive among the people. It is interesting to note that, in the beginning, we used to tell people that, as one of the ways to encourage them to donate blood voluntarily, they would be checked for infectious disease markers through donation and they would know on their infection status! This was just aimed to promote interest among prospective donors those days. This practice was soon ceased once we realized that it is not in good spirit that a donor donates blood just to know the status for any positive infectious markers. The idea of directed/replacement donations was also put in force to meet the demand for the blood units. With this approach in the procurement of blood, we were self-sufficient to meet the demand and, by July 1991, we completely stopped the import of blood as we had generated an overwhelming enthusiasm among all walks of life, which is reflected by the statistics on blood collection by the Ministry's blood banks as displayed in Table 1.

**Table 1:** Statistic on blood collection by the blood banks of the ministry of health in the initial period between 1990 and 1993

Year	*n* Unit collected	Yearly increment	Increment (%)^a^
1990	7000	-	
1991	7155	155	2.21
1992	8140	985	13.77
1993	10133	1993	24.48

a % increment as to previous year collection

## Blood Banking Strategy

We applied the principle of the division of labor. The activity of Blood Transfusion Services was divided among the blood banks of the two major hospitals in the city of Muscat, viz. the Khoula Hospital and the Royal hospital. The responsibility of procurement of blood through organized outdoor camps was entrusted to the blood bank at the Khoula hospital while processing on collected blood units, including screening for infectious markers, labeling, storage, and distribution as finished product, was rested with the blood bank at the Royal hospital. Blood collection from the replacement by the relatives was, however, continued at the individual blood banks of the hospitals.

Recruitment and retention of voluntary blood donors for adequate and safe blood supply was a herculean task for us. We put our efforts on donors' motivation through education by creating awareness among the people on blood donation. These efforts paid us back in terms of eliminating the practice of “payment” toward donation. However, we continued to give a token gift as an incentive for their generosity toward blood donation. Over a period, the awareness on voluntary blood donation was so intensive and successful that eventually we had stopped giving any token gift and made it a purely voluntary nonremunerated program. We were very happy at this stage that the program had made us self-reliant on local voluntary blood donation freeing us from menace of importing blood. While recognizing our efforts, the Ministry had provided with facilities to have our own independent existence by allocating a porta-cabin kind of structure with our own staff to run the blood center in the neighborhood of the Khoula Hospital in Wattayah locality at Muscat.

The National Blood Transfusion Services (NBTS) was established in 1993 under the umbrella of the Ministry of Health. A committee was formed to look into the affairs related to the blood transfusion services. The members of this committee were professionals from the hospital services not only of the Ministry of Health but also came from the institutions of the other Ministries such as the University Hospital, the Armed Forces Hospital, etc. A representative from the WHO office in Oman was also a member of the NBTC. The committee had the responsibility to formulate and implement policies regarding blood transfusion practices in the country. A provision for subcommittee on *ad-hoc* bases was made as and when it is required. For example, currently we have formed a technical subcommittee to update and revise the Blood Transfusion Technical Manual. Once this task is accomplished, some other manuals on the management of blood transfusion services will be updated by such an *ad-hoc* subcommittee.

The Department of Blood Services as a separate unit was established by the Ministry in 1995 and 3 years later it moved into new independent premises at Bausher where the central blood bank is located. The space at the new premises had allowed us to implement our program more efficiently. A well-equipped laboratory with proven technologies have allowed us to give the expected highest possible standards of donor care and donor blood screening program to ensure to provide safe blood in an efficient manner. The round the clock service was commenced.

## Concept of Regional Blood Banks

**Figure 1:**
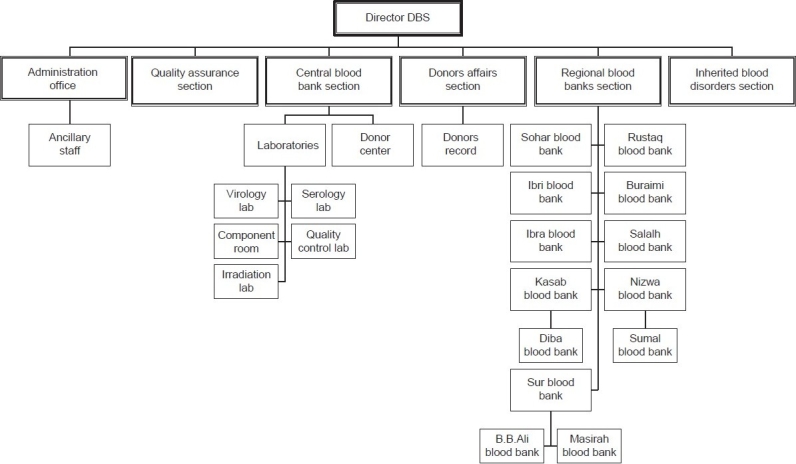
Organization chart

In order to disseminate the idea of voluntary blood donations at the regional level, the Regional Blood Banks, already in function at hospitals in different regions, were put under the direct care of the Department of Blood Services by 1996. There are 14 such blood banks, including the central blood bank, in operation throughout Oman. These regional centers are at BB Ali, Buraimi, Dibba, Ibra, Ibri, Khasab, Masirah, Muscat (Central Blood bank), Nizwa, Rustaq, Salalah, Sohar, Sumail, and Sur [Figure 1]. The main activity of the regional blood banks remained to meet emergency-to collect blood from the relatives and process-screen for HIV, Hepatitis B using rapid test (later on replaced by ELISA), and cross-match. In further development, screening for anti-HCV for hepatitis C infection using commercial kits was implemented at later point of time. In mid-1999, the ISBT labeling system was adapted. Some of these blood banks may or may not have any facility for storage. The Department's central blood bank works in cohesion with these regional blood banks and shoulders the responsibility in supply of blood/components on regular basis. The Regional Blood Banks were asked to adopt the policy of maintaining a reasonable stock of screened blood to meet the requirements of their own hospital services. This is a way to reduce the burden on the Central Blood Bank but still the Central Blood Bank supplies the regional hospitals with safe blood/blood components as per their request. Some regional blood banks are provided with equipment to prepare platelets locally, with an expectation to meet their local demand. The Department of Blood Services has now introduced the server to interconnect the computers at all the regional blood banks to handle a large database created on the donors' records.

Under the direct control of the Department of Blood Services, entire blood bank network was established through centralization of blood banks. The value of centralized transfusion services has been demonstrated by improved resource management and cost reduction and enhanced productivity. By connecting all blood banks through the computer server, the services are efficiently managed. Centralization of donor database has helped stream-lining the donor recruitment strategies as well as getting rid of any duplication in services. It also helped in standardization of laboratory procedures so as to have uniform practice throughout.

**Figure 2:**
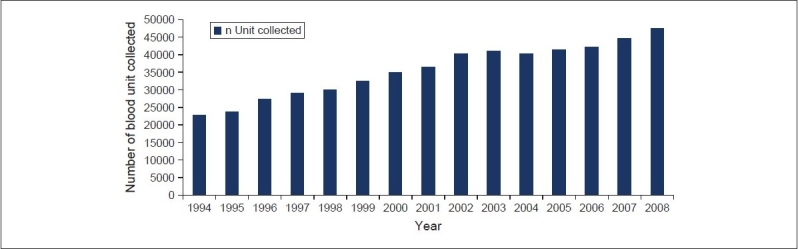
Year-wise blood collection

The statistics on blood donation movement evidently suggest that a modest collection of 7000 blood units in the year 1990 has reached to a hefty figure of over 47,000 by 2008. During the intervening period, blood banks have, almost always, witnessed some yearly increment in the number of blood units collected. The year-wise blood collection data are displayed in Figure 2.

## Current Scenario

Figure 2 shows the organization chart depicting Department of Blood Services of the Ministry of Health, Oman.

Table 2 displays the statistics on blood donations and the donors' profile in blood banks in Oman for the last 3-year period from 2006 to 2008. The data include the blood collection in various blood banks in Oman including those of the Ministry of health and hospitals of the University, the Royal Oman Police, and the Royal Oman Army.

**Table 2:** Statistical data on blood collection and demography of the donors at blood banks in Oman

Year	No. of donors	Male	Female	Voluntary	Family replacement	First time	Repeated
2006	42211	39367	2844	30996	11215	19462	22749
%		93.26	6.74	73.43	26.57	46.11	53.89
2007	44611	41672	2939	32883	11728	20721	23890
%		93.41	6.59	73.71	26.29	46.45	53.55
2008	47478	44425	3053	35096	12382	23249	24229
%		93.57	6.43	73.92	26.08	48.97	51.03

A steadily increased pattern in collection of blood units is obvious as in the year 2007, 2400 (i.e., 5.69%) more blood units were collected when compared to the collection in the previous year of 2006. Similarly, there was an increase of 2867 blood units in 2008 that gives an increment of 6.43% as compared to the collection in previous year, that is, 2007.

The data on gender profile show that while the most blood units were donated by the male donors, the female donors constitute only 6-7% of the blood donors-the trend that is consistence with the figures obtained in many developing countries.

While majority of the donors were voluntary donors, about one-fourth of the donors were replacement donors from the family members of the recipients.

Nearly half of the donors donated by first time and remaining half were the repeat donors; the trend clearly indicates toward the efforts being made by the blood banks to retain their donors.

## Voluntary Non Remunerated

**Figure 3:**
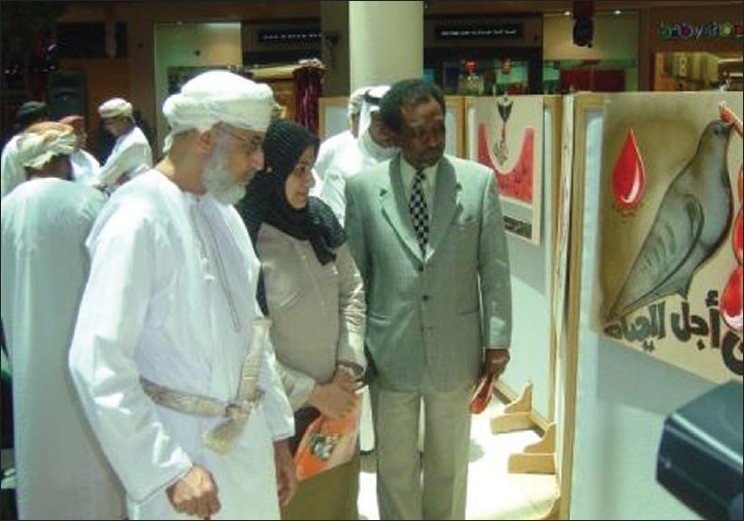
Poster visit

**Figure 4:**
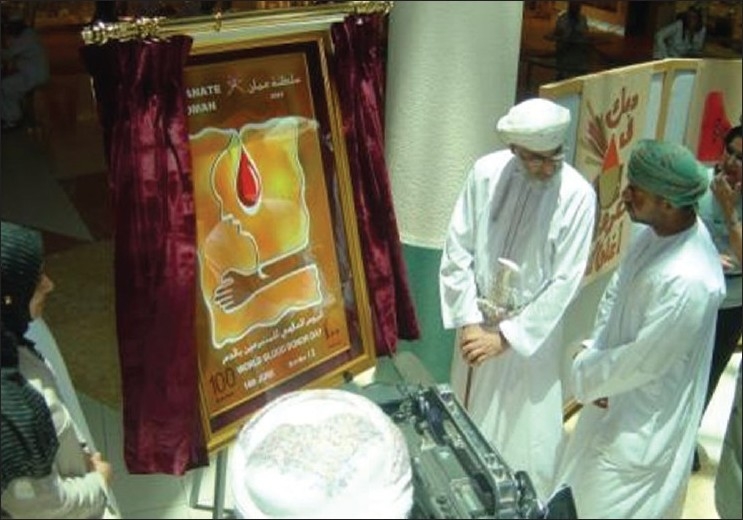
World donor day opening

**Figure 5:**
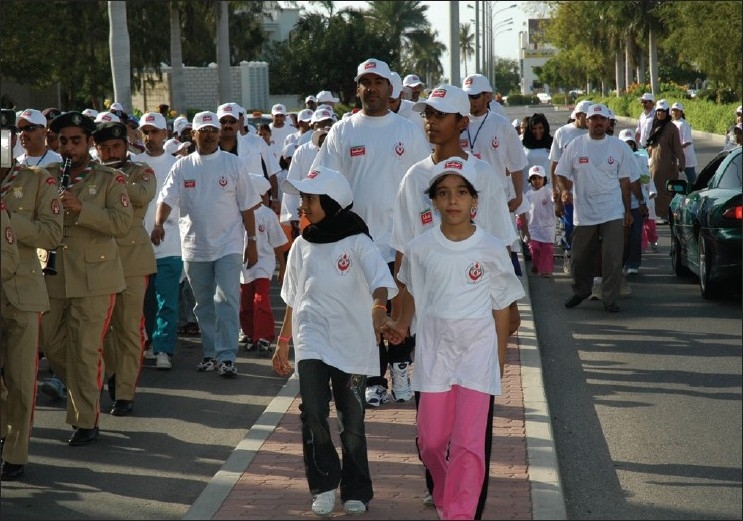
Marathon 1

**Figure 6:**
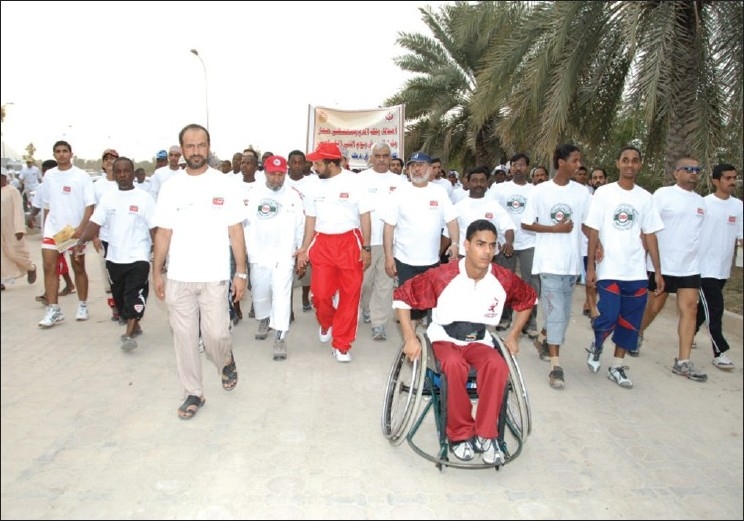
Marathon 2

**Figure 7:**
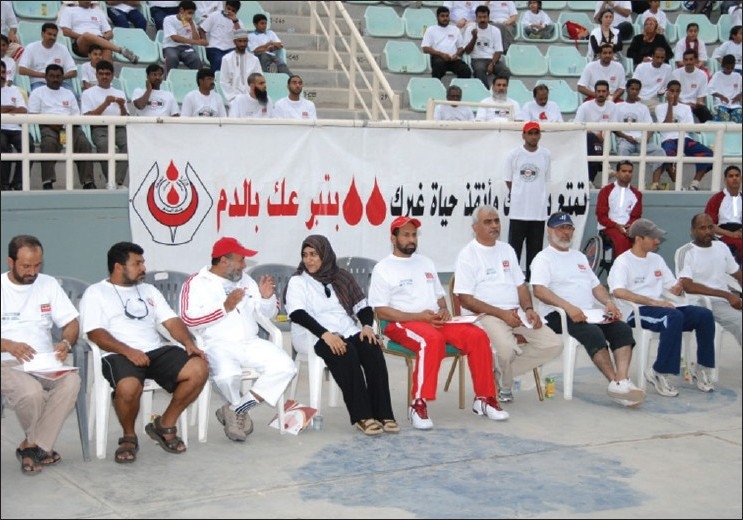
Marathon 3

**Figure 8:**
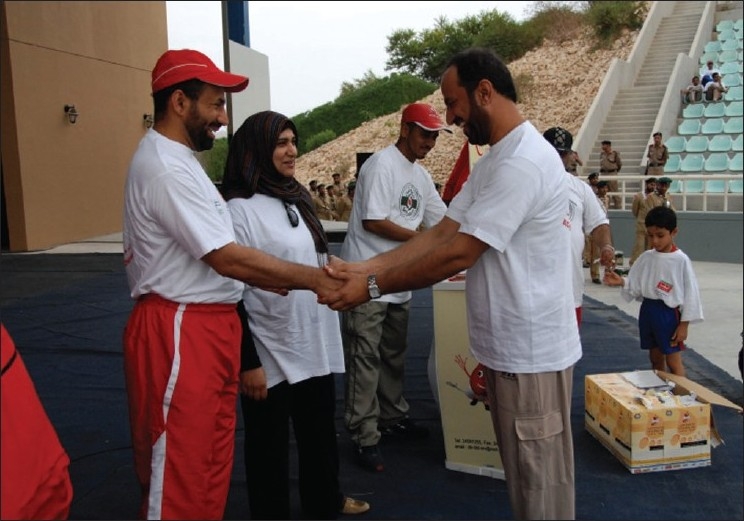
Marathon end

**Figure 9:**
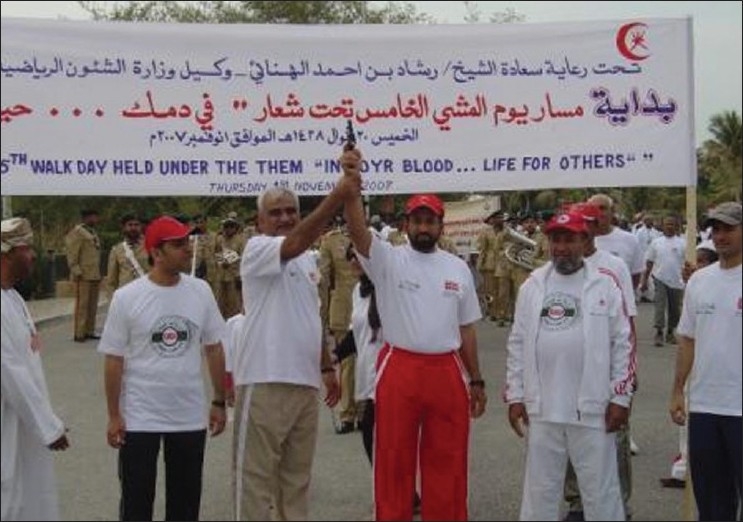
5^th^ walk day

**Figure 10:**
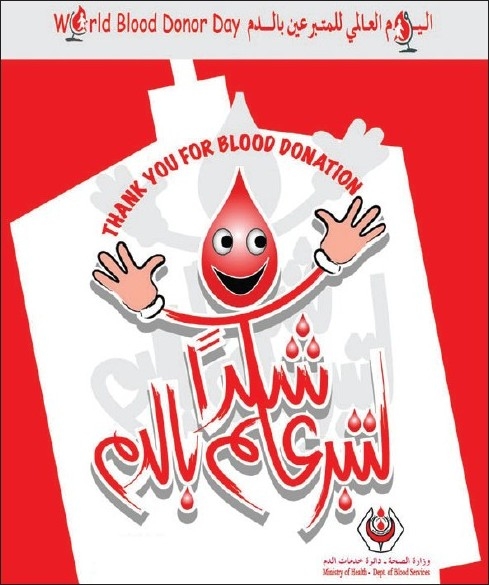
Blood poster

**Figure 11:**
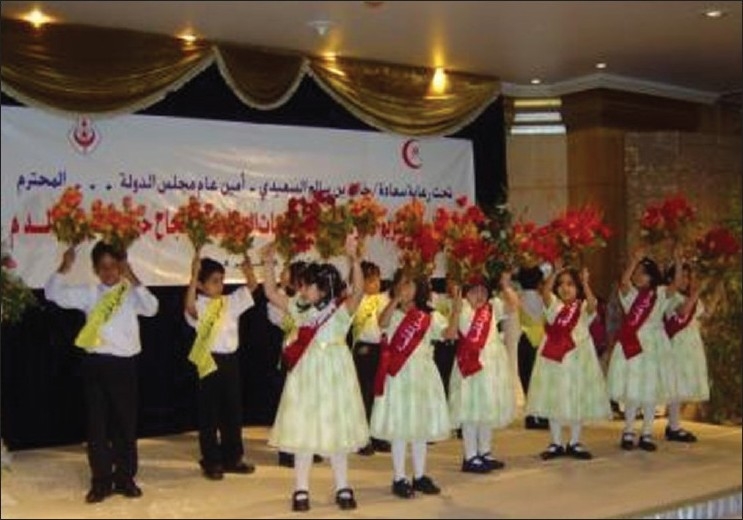
Cultural program donors' day 1

**Figure 12:**
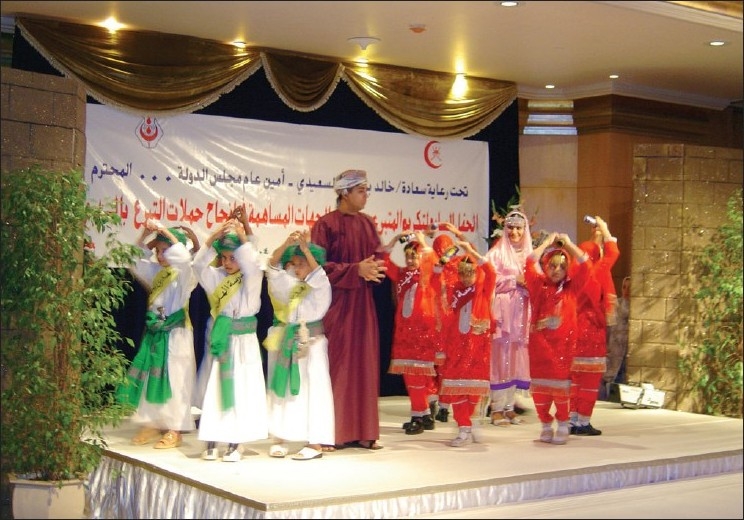
Cultural program on donors' day

**Figure 13:**
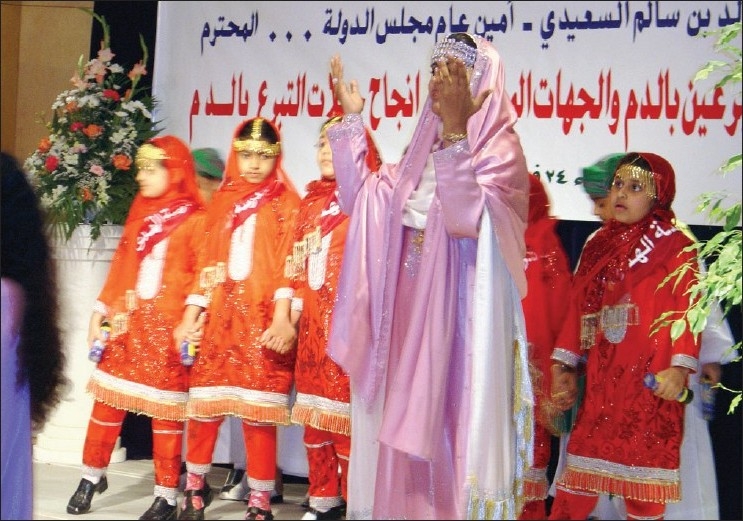
Cultural program donors' day 3

**Figure 14:**
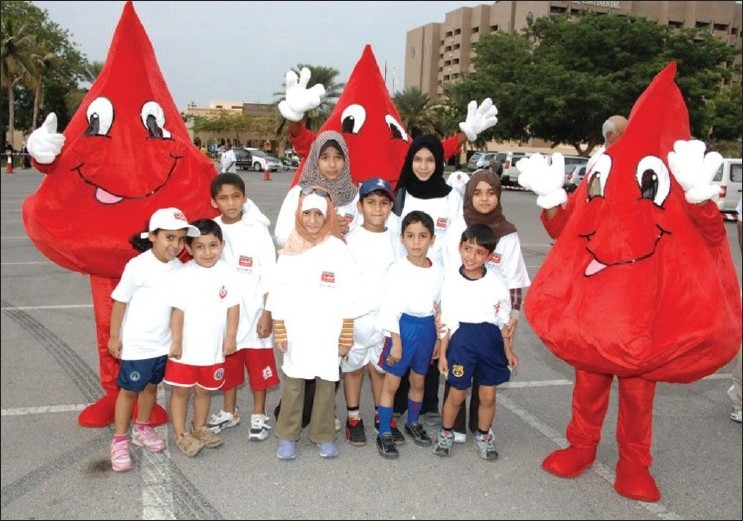
Damdoom

**Figure 15:**
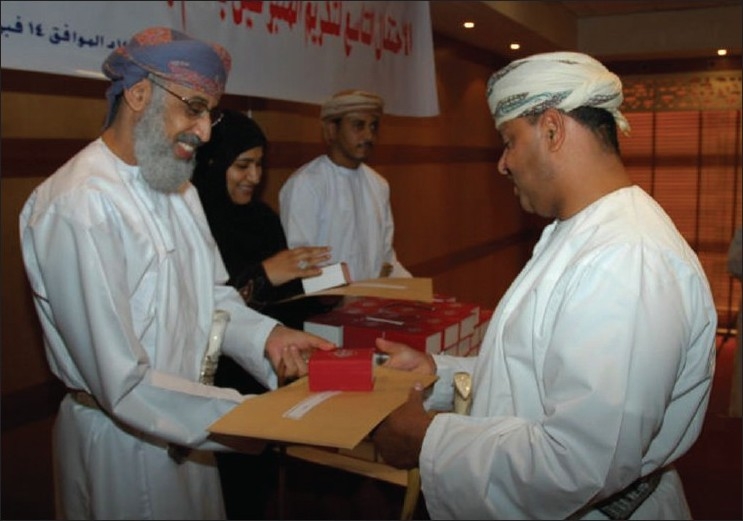
Donors' felicitation 1

**Figure 16:**
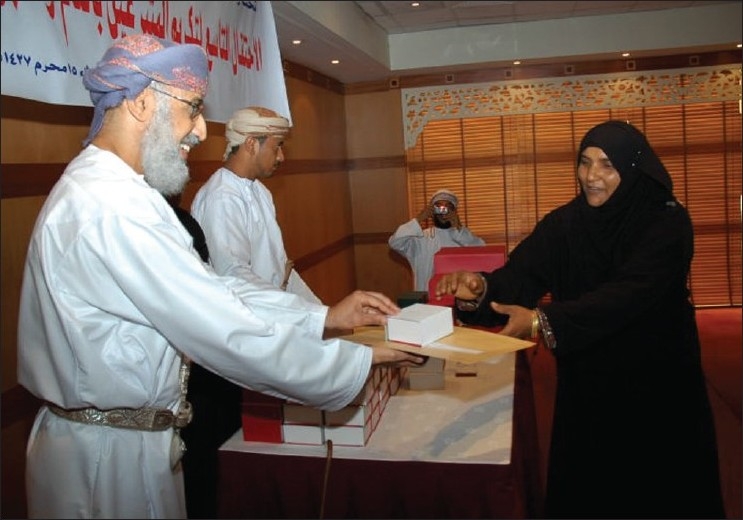
Donors' felicitation 2

Campaign as to promote the voluntary, nonremunerator program to augment blood collection is a backbone to such a kind of activity. In order to create awareness on blood donation, the Department of Blood Services has been actively engaged in conducting various activities with sole aim to educate and bring general awareness among the local population about blood donation. The World Health Day on 7^th^ April, 2000, was dedicated to blood donation with the slogan “Safe Blood Starts with Me.” The Department of Blood Services arranged to issue 30,000 telephone cards with this slogan embossed as one of the steps of disseminating the general awareness about blood donation to the masses. The Department of Blood Services has decided to celebrate the annual blood donor day. We used to have this celebration in the month of February every year, but the date was flexible depending on an availability of the dignitary to preside over the function. However, now we have fixed the date that coincide with the World Blood Donors Day, that is, June the 14^th^ every year [Figures 3 and 4]. On this occasion, Department of Blood Services organizes manifold activities like marathon walk involving youth [Figures 5–9], cultural programs involving children [Figures 10–14] and felicitating individual donors or organizations with remarkable contribution by blood donation Figures 15–16. Pictures/photographs taken on such occasions and being displayed here speak themselves on overwhelming enthusiasm shown by the members of the society in general. The introduction of Blood Donor Felicitation Day has influenced a lot many people who are now coming forward to donate on regular basis. The idea has thankfully influenced the regional blood banks to honor their donors as well.

## State of Art Technology

**Figure 17:**
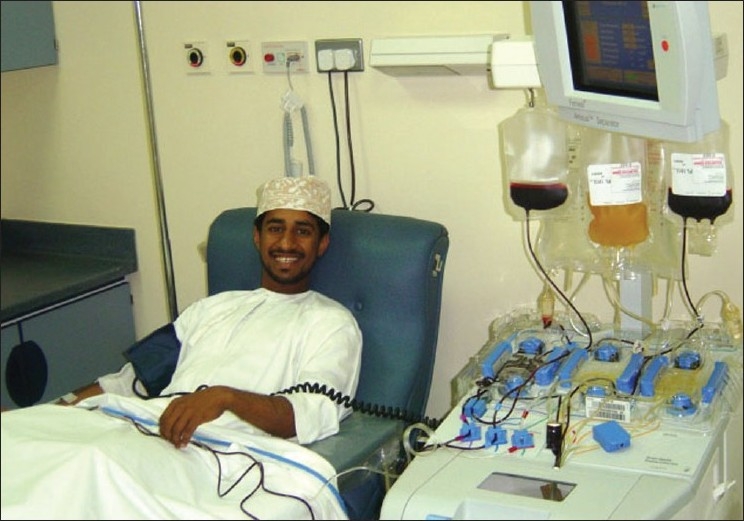
Apheresis donation

Donors' blood grouping and antibody screening is carried out with state of art technology of gel-techniques using commercial reagents/equipment. Antibodies to red cell antigens are screened with two sets of commercial screen cells and antibody detected, if any, is identified using a commercial set of 11 red cell panel. Screening of blood donors for transfusion transmitted infections markers like HBsAg, anti-HBc, anti-(HIV-1 and HIV-2), and anti-HCV is done using commercial kits of Axim automation device. For syphilis, we use TPHA. Almost all the blood units collected are separated into blood components like red cells, fresh frozen plasma, cryoprecipitates, and platelets. Single donor platelets are now possible to collect by apheresis [Figure 17]. Apheresis donors are usually recruited from the registered WB donors. Plateletapheresis donors may donate more frequently than WB donors, with informed consent but must meet all other donor criteria, particularly of having a minimum of platelet count of 150,000/μL. Since Jan 2005, all the platelet units collected at Central Blood Bank are processed as to leucoreduced and irradiated using gamma-irradiator device installed.

National Reference Serology Lab is established at the Central Blood Bank, Muscat, and deals exclusively with complexities in serological problems related to anomalous blood grouping and difficulty in cross-matching. Besides, we participated in EQAS programs, both locally through Public Health Laboratory in Muscat and internationally (UKNEQAS).

## Training Programs

The Department of Blood Services organizes periodic training programs for the staff working in the regional blood banks in Oman. The areas of training include in organizing outdoor blood donation camps, targeting and motivating blood donors, and mobile blood collection. Besides, the department also organizes annual CME in the form of a 2-day workshop on immunohematology to refresh the knowledge of the technical staff of the regional blood banks.

## Conclusion

Sultanate of Oman can now boast on having one of the modern blood transfusion services in the region. It has created advanced facilities including providing irradiated blood in need of hours. However, this has not come without the hard work of those associated with the service and an excellent moral and material support rendered by the Government. This report summarizes an account on how the blood transfusion service took its present shape over a period of three decades.

